# Is a Low Incidence Rate of Ventilation Associated Pneumonia Associated with Lower Mortality? a Descriptive Longitudinal Study in Iran

**Published:** 2018-02

**Authors:** Arezoo Chouhdari, Shervin Shokouhi, Farshid Rahimi Bashar, Amir Vahedian Azimi, Seyed Pouzhia Shojaei, Mohammad Fathi, Reza Goharani, Zahra Sahraei, Mohammadreza Hajiesmaeili

**Affiliations:** 1 Skull Base Research Center, Loghman Hakim Hospital, Shahid Beheshti University of Medical Sciences, Tehran, Iran; 2 Department of Infectious Disease, Loghman Hakim Hospital, Shahid Beheshti University of Medical Sciences, Tehran, Iran; 3 Department of Anesthesiology, School of Medicine, Hamadan University of Medical Sciences, Hamadan, Iran; 4 Trauma Research Center, Nursing Faculty, Baqiyatallah University of Medical Sciences, Tehran, Iran; 5 Department of Critical Care Medicine, Loghman Hakim Hospital, Shahid Beheshti University of Medical Sciences, Tehran, Iran; 6 Anesthesiology and Critical Care Research Center, Shahid Beheshti University of Medical Sciences, Tehran, Iran; 7 Department of Clinical Pharmacy, School of Pharmacy, Shahid Beheshti University of Medical Sciences, Tehran, Iran; 8 Department of Anesthesiology and Critical Care, Anesthesiology Research Center, Loghman Hakim Hospital, Shahid Beheshti University of Medical Sciences, Tehran, Iran

**Keywords:** VAP, Incidence, Attributable mortality rate, Case fatality rate, ICU, Cost

## Abstract

**Background:**

Ventilator-Associated Pneumonia (VAP) is an important cause of morbidity and mortality in patients admitted to Intensive Care Unit (ICU). The current study conducted to estimate VAP incidence, attributable mortality and case fatality rate, cost, so related factors can affect the outcome in patients.

**Materials and Methods:**

In this descriptive longitudinal study, demographic, clinical and para-clinical data were collected and attributable mortality and case fatality rate was estimated. Multivariable analysis was done to predict the possible risk factors on the outcome of VAP patients. Also, patients’ survival curve was plotted based on their length of ICU stay. Finally, the additional cost due to VAP in ICU was estimated.

**Results:**

Totally, 8% ICU admissions were affected by VAP and 4% expired during the ICU stay. Further, the attributable mortality rate of VAP was high as compared with standard mortality rate. The most case fatality rate was for Acinetobacter sp. (n=17 60.7%). In multivariable logistic regression analysis, age greater than 40 years, more than 96 hours mechanical ventilation and uncontrolled diabetes mellitus were predictor factors of higher mortality. Inverse association between survival time and ICU length of stay was reported. Finally, the additional cost of VAP was estimated of about 700 US$ per patients.

**Conclusion:**

According to the results, strategies to prevent mortality by reducing the duration of ventilation and ICU length of stay should be performed. Also, mandatory fees for the family and the healthcare system should be planned.

## INTRODUCTION

Hospital-acquired pneumonia, is the second most common infection after urinary tract infections in the hospital and includes about 30% of all nosocomial infections. Ventilator Associated Pneumonia (VAP) is a subset of nosocomial pneumonia occurring 48 hours or more after undergoing Mechanical Ventilation (MV) via endotracheal or tracheostomy tube (1,2). The most important micro-organisms responsible for infection are *Staphylococcus aureus* (*S. aureus*), *Pseudomonas aeruginosa* (*P*. *aeruginosa)*, and *Enterobacteriaceae*, but etiologic agents widely differ according to the population of patients in an Intensive Care Unit, duration of hospital stay, and prior antimicrobial therapy. VAP is a serious, common and costly complication ranking first among nosocomial infections in ICU. Prevalence of VAP is between 9–27% and this mortality rate has been reported 30–70%. In some studies, the mortality rate of VAP is 16 to 20% (3–7). The risk of VAP in patients who are receiving MV increase 1–3% for every day of hospitalization. Different studies estimated the prevalence of VAP, 10.2% to 32% in 1000 person (5). Intubation, alone as a risk factor for pneumonia in hospitalized patients, is considered (6). There are several factors that increase the frequency of VAP including length of hospital stay, underlying diseases such as central nervous system, gastrointestinal tract infections, and multiple trauma, chronic lung disease, the supine position of the patient, surgery, chronic renal failure and the use of steroids (8–10). Also, unconsciousness, tracheotomy, multiple intubations during hospitalization, emergency intubation and nasogastric tube also affect the incidence of VAP (10,11). Although many studies indicate the low incidence of VAP following proper hand washing and use of protective gloves, but study on risk factors for mortality is limited.

Therefore, the aim of this study was to determine the incidence and mortality rate of VAP and related factors in a tertiary hospital in Iran.

## MATERIALS AND METHODS

In this descriptive longitudinal study 1221 patients who were admitted to the multi-center ICU of Loghman Hakim Hospital, Tehran, Iran during one year from March 2016 to March 2017 were evaluated. All patients who fulfilled the diagnostic criteria, confirmed by Center for Diseases Control and Prevention (CDC) (12–15), were enrolled and followed up within 30 days, (Table 1). Selected patients had stayed in ICU for at least 2 days and received MV within 48 hours after ICU admission. If the criteria for VAP diagnosis were not complete or patient had MV for less than 48 hours, they were excluded.

**Table 1.** VAP definition

VAP two-stage Definition	
Clinical VAP Definition	Microbiological VAP Definition
Radiological changes: A chest x-ray or computed tomography scan suggestive of pneumonia (2 or more required for patients with underlying cardiac or pulmonary disease) and at least one of the following Systematic inflammation including white cell count of >10000/mm^3^ or <4000/mm^3^ Or Temperature >380C with no other cause and at least one of the following (Two required if microbiology is by qualitative endotracheal aspirate culture or if cultures are negative). Clinical pulmonary signs: New onset of purulent sputum or change in character (color, odor, consistency or quantity) Or a cough or dyspnea or tachypnea Or Auscultatory findings (rales, bronchial breathing, ronchi, wheeze) Or Worsening gas exchange (including desaturation, increasing FiO_2_ or ventilator requirement.	Bacteriologic confirmation: Either Positive quantitative culture from minimally contaminated lower respiratory tract samples (including growth at>10^4^ CFU/ml from bronchoalveolar lavage (BAL) cultures) Or positive blood cultures without another source, positive pleural fluid culture, pleural or pulmonary abscess with culture-positive needle aspirate, histological evidence of pneumonia, non-culture methods of detection (legionella antigen, viral PCR) Or The positive qualitative culture of endotracheal aspirates or non-directed mini-BAL, negative cultures meeting clinical criteria above is recorded as “clinical VAP”.

After VAP diagnosis, a data collecting form that was designed with collaboration of ICU, Infectious disease and Preventive medicine specialists, was filled by trained experts at the bedside of patients. This form included demographic information, type of primary admission (trauma, medical or surgical), reasons of ventilation, place of ventilation (prehospital, ICU, emergency room, hospital wards and operating room), duration of MV, Glasgow Coma Scale (GCS), length of hospital and ICU stay, comorbidity (uncontrolled diabetes mellitus, hypertension and underlying heart diseases) and laboratory tests.

To measure and predict prognosis of patients admitted to multicenter ICU the APACHE II score at first day of admission was used. According to standard table, mortality rate in score 0–15, 16–19, 20–30, and over 30 was 10, 15, 35 and 75%, respectively (15). According to this explanation, attributable mortality rate was estimated. Furthermore, we assessed the organisms leading to VAP through microbial cultures and Case Fatality Rate (CFR) [proportion of deaths within a designated population of “cases” (people with a medical condition), over the course of the disease] estimated based on results of microbial culture.

Association between variables under study and patients outcome within 30 days was evaluated by Chi 2 and Fisher exact tests, independent t-test and Mann-Whitney U tests. Adjusted odds ratio was calculated in multivariable logistic regression to predict outcome according to covariables in this study. Also, survival curve was plotted. The protocol for the study was approved by the Ethics Committee of the Shahid Beheshti University of Medical Sciences. Total analysis was executed by spss19 and level of significance for all tests was considered as p. value <0.05.

## RESULTS

From 1221 hospitalized patients in multi-center ICU (general, surgery, emergency, and neurosurgery) during 2016–2017 in Loghman Hakim Hospital, 100 patients (8%) fulfilled the inclusion criteria of VAP diagnosis within 30 days. The basic characteristics of patients are shown in table 2. In this study APACHE II score of patients was 44.8, 52, and 70.6%, respectively. An attributable mortality rate of patients affected by VAP was 34.8, 37, and 35.6%, respectively (Table 3). Case fatality rate in different types of micro-organism is indicated in table 4.

**Table 2.** Characteristics of patients with VAP

**Variables**		
**Age, years**		52.45(±21.004)
**Gender**	Male	69(69)
	Female	31(31)
**BMI**		26.20(±7.09)
**Type of primary admission**		
Trauma		15(15)
Medical		39(39)
Surgical		46(46)
**Place of Intubation**		
Pre-hospital		10(10.2)
ICU		23(23.5)
Emergency department		27(27.6)
Operating room		30(30.6)
Hospital wards		8(8.2)
**Reason of ventilation**		
HAP^1^ including aspiration		2(2)
Alternation level of consciousness		48(48.5)
Sepsis, Septic shock		0(0)
Pulmonary edema		4(4)
Asthma/COPD		2(2)
Cardiac arrest		2(2)
Surgery		36(36.4)
Community-Acquired Pneumonia		5(5.1)
Respiratory failure of unknown etiology		0(0)
**Bacterial strains found in cultures**		
**Acinetobacter sp**		28(36.8)
**Enterobacteriaceae**		
Klebsiella pneumoniae		19(25)
Escherichia coli		6(7.8)
Enterpbacter sp		2(2.6)
Citrobacter diversus		1(1.4)
**Staphylococcus aureus**		12(15.8)
**Psudomonas aeruginosa**		6(7.9)
**Contamination**		2(2.7)
**Length of ICU stay**		15.08(±13.10)
**Length of hospital stay**		22.46(±16.48)
**Underlying diseases**		
Uncontrolled DM		17(17)
HTN		93(93)
Heart diseases		20(20)
**ICU Discharge Alive**		
Yes		60(60)
No		40(40)
**Duration of ventilation(hours)**		196.74(±192.07)

1 Hospital-acquired pneumonia

* Data presented as mean (±SD) or No(percent%)

**Table 3.** Attributable mortality rate in VAP patients according to APACHE II score system

**APACHE II score**	**Frequency (%)**	**Mortality Rate**	**Standard Mortality Rate**	**Attributable Mortality Rate**
0–15	58 (58%)	26(44.8%)	10%	34.8%
16–19	25 (25%)	13(52%)	15%	37%
20–30	17 (17%)	12(70.6%)	35%	35.6%
>30	0 (0%)	-	75%	-

**Table 4.** Case fatality rate in different types of micro-organism in patients with VAP

**Result of BAL culture**	**Case fatality rate**	**Result of BAL culture**	**Case fatality rate**
Acinetobacter sp	17(60.7%)	Pseudomonas aeruginosa	3(50%)
Klebsiella pleumoniae	10(52.6%)	contamination	1(50%)
Escherichia coli	3(50%)	Staphylococcus aureus	5(41.6%)
Enterobacter sp	1(50%)	Citrobacter diversus	0(0)

By chi 2 univariable analysis, there was a significant difference between place of ventilation (pre-hospital, ICU, emergency room, hospital wards, operating room) and expiration in ICU (p=0.01). So that, 40% of patients expired in ICU had been ventilated in ICU. Also, there was a significant difference between the duration of ventilation (hours) and expiration in ICU (268.21±212.389 vs. 147.93±162.028), (p=0.01). But, there was no significant difference between age, sex, Body Mass Index (BMI), type of primary admission, reason of ventilation, duration of ventilation (hours), micro-organisms found in Broncho-Alveolar Lavage (BAL) culture, tracheostomy, APACHE II score, Glasgow Coma Scale/Score (GCS), uncontrolled diabetes, hypertension, underlying heart diseases and ICU expiration, statistically (p<0.05).

**Figure 1.**
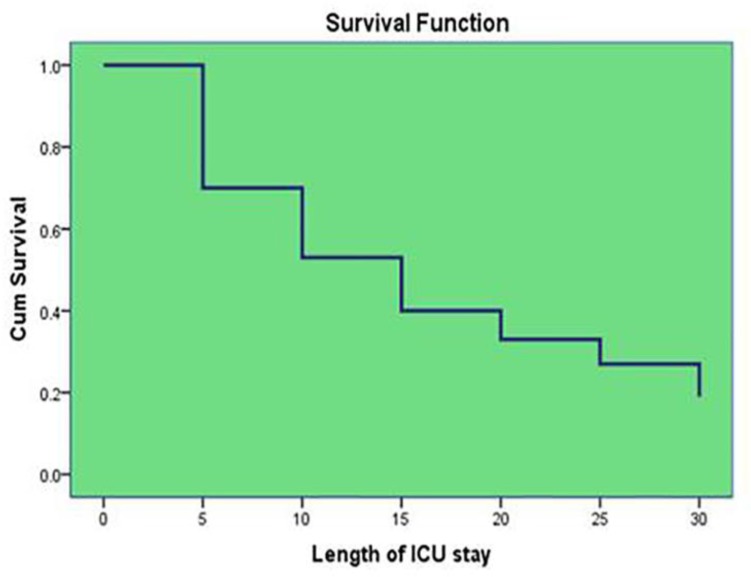
Hospital survival among patients with VAP

The outcome in VAP patients according to covariables was estimated with multivariable logistic regression. Age greater than 40 years (OR: 6.7,95%CI 1.1–39.1, p=0.03), more than 96 hours of MV (OR: 1.5,95%CI 1.01–23.4, p=0.01) and uncontrolled diabetes mellitus (OR: 1.07,95%CI 1.01–3.9, p=0.03) were predictor factors of mortality (Table 5). Finally, survival time reduces with increased ICU length of stay (Figure 1). The mean added pay out of pocket due to more length of VAP ICU stay was estimated as 700 US$ per patients.

**Table 5.** Multivariable analysis for prediction of mortality in patients with VAP

**Variables**	**Reference group**	**OR(95%CI)**	**P. value**
**Sex**	Male	5.8(0.7–46.2)	0.09
**Age**	40y≥	6.7(1.1–39.9)	0.03*
**Reason of ventilation**			
HAP including aspiration	Decrease of	0.1(0.07–2.1)	
Cardiac or pulmonary problem	Consciousness	0.05(0.01–3.5)	0.1
Surgery	level	0.2(0.1–2.5)	
**Mechanical ventilation**	96h ≥	1.5(1.01–23.4)	0.01*
**Type of patient**			
Medical		3.2(0.2–44)	
	Trauma		0.3
Surgery		1.2(0.1–12.7)	
**BMI**			
25–30		3(0.8–9)	
	19–24		0.1
Over 30		0.1(0.05–0.1)	
**GCS**	≥7	0.2(0.2–2.5)	0.5
**APACHE II score**			
16–19		0.6(0.3–4)	0.4
	0–15		
20–30		0.3(0.1–0.5)	0.3
Over 30		0.3(0.1–0.5)	0.2
**Place of ventilation**			0.09
ICU		0.1(0.01–2.8)	0.2
Emergency department	Pre-hospital	2(0.2–14.4)	0.4
Operating room		0.2(0.04–1.7)	0.1
Hospital wards		0.9(0.1–6.2)	0.9
**Length of ICU stay**	7days≥	1.02(0.2–3.9)	0.9
**Uncontrolled DM**	No	1.07(1.01–3.9)	0.03*
**Underlying heart diseases**	No	1.4(0.3–6.9)	0.6
**HTN**	No	0.3(0.02–7.3)	0.5

Significance statistically shown with ^*^

## DISCUSSION

In this study, the incidence rate of VAP in ICU was 8% in one year among 1221 patients admitted in ICU, while other studies estimated VAP about 8% to 28% (16–18). Our estimation is low as compared with other studies. The reason for this low rate can be compliance with prevention strategies such as basic practices to prevent VAP in adult patients including: decrease duration of MV and length of ICU stay, avoidance of intubation if possible, minimize sedation, maintain and improve physical conditioning, minimize pooling of secretions above the endotracheal tube cuff, elevate the head of the bed and maintain ventilator circuits (18,19).

Despite the low incidence, the death rate was almost high (41%). Some study estimated the frequency of VAP in care units specifically, 10 to 65% and the rate of mortality from 20 to 70% (7–9,20). Overall, rates of pneumonia are considerably higher among patients in ICUs compared with those in hospital wards, and the risk of pneumonia is increased 3 to 10 fold for the intubated patient receiving MV. Nevertheless, VAP is not always ‘associated’ with the ventilators but with the artificial airways (endotracheal tubes and tracheostomies cannulae) as well (21–23).

In this study according to APACHE II score, the VAP attributable mortality rate in ICU cases was about 34.7–37%, but this rate is variable and relies heavily on the underlying medical illness (20). Reports indicated mortality rate changes from 24 to 50%. Sometimes total mortality rate reaches 76% in some specific settings or when lung infection is caused by high-risk pathogens (7–9,17).

In this survey, univariable analysis with chi 2 demonstrates a significant difference between ICU ventilation and expiration in ICU (p=0.01). So, there was a significant difference between the duration of ventilation (hours) and expiration in ICU (268.21±212.389 vs. 147.93±162.028), (p=0.01). Odd ratio estimations in multivariable logistic regression analysis predicted age more than 40 year, MV higher than 96 hours and uncontrolled diabetes mellitus were related to expiration in VAP patients in 30 days. Huang et al. showed that an APACHE II score >27 at VAP onset was an independent and early predictor of the mortality (21). Inchai et al. demonstrated identified the prognostic indicators that included co-morbid malignancy (hazard ratio [HR]=1.60; 95z confidence interval [CI] 1.02–2.42; P=0.040), septic shock (HR=2.51; 95% CI, 1.60–4.00; P<0.001), Simplified Acute Physiology Score II>45 (HR=1.62; 95% CI, 1.03–2.56; P=0.041), and Sequential Organ Failure Assessment score>5 (HR=3.40; 95% CI 2.00–5.81; P<0.001), (22). In published article by Melsen et al. in 2013, the highest cumulative risks for dying from VAP were noted for surgical patients (2·97, 95% CI 2·24–3·94) and patients with midrange severity scores at admission (cumulative risks of 2.49 [1.81–3.44]) with APACHE scores of 20–29 and delayed inappropriate empirical antibiotic treatment (HR=2.23; 95z CI, 1.12–4.45; P=0.022) most died (23).

In general, early VAP is caused by micro-organisms that are sensitive to antibiotics, whereas late-onset VAP is caused by multi-drug resistant pathogens and are more difficult to treat (24,25). Although in this study the most case fatality rate was related to *Acinetobacter* spp, in one other study, *P*. *aeruginosa* (24.4 %), S. aureus [20.4 % of which > 50 % Methicillin Resistant Staphylococcus aureus (MRSA)] was associated with higher prevalence and mortality (26). Nevertheless, host factors such as the severity of underlying disease, previous surgery, and antibiotic therapy have all been implicated as risk factors for increasing VAP mortality (27–29). Also, cumulative survival had inverse relation to length of ICU stay that was confirmed by another study (6,29–32).

This survey, like most studies, has limitations such as the unavailability or incompleteness of certain data, recording of patient’s vital signs by unskilled nurses with different precisions and some other restrictions. Although incidence rate and cost of length of ICU stay due to VAP is lower than many studies (24,30,33,34); however, due to high mortality and financial burden on the family and the healthcare system, practical steps should take for the prevention this disease.
